# Prognostic Factors of Mortality and Clinical Characteristics of Patients Surgically Treated for Blunt Liver Injury—A Single-Center Experience

**DOI:** 10.3390/jcm15176873

**Published:** 2026-09-04

**Authors:** Bartłomiej Białas, Maciej Rybicki, Piotr Tomasz Arkuszewski, Michał Kusiński

**Affiliations:** 1 Ward of Endocrine, General and Oncological Surgery, Faculty of Medicine, Medical University of Lodz, M. Kopernik Provincial Multispecialist Centre for Oncology and Traumatology in Łódź, 91-513 Lodz, Poland; bartlomiej.bialas@stud.umed.lodz.pl (B.B.); maciej.rybicki@stud.umed.lodz.pl (M.R.); 2 Plastic, Reconstructive and Aesthetic Surgery Clinic, Institute of Surgery, Medical University of Lodz, 90-419 Lodz, Poland; 3 Department of Biomedicine and Experimental Surgery, Faculty of Medicine, Medical University of Lodz, Narutowicza 60, 90-136 Lodz, Poland; piotr.tomasz.arkuszewski@umed.lodz.pl; 4 Department of Endocrine, General and Oncological Surgery, Faculty of Medicine, Medical University of Lodz, M. Kopernik Provincial Multispecialist Centre for Oncology and Traumatology in Łódź, 91-513 Lodz, Poland

**Keywords:** blunt liver trauma, road traffic accident, falls from height, predictive factors, trauma mechanisms, Glasgow Coma Scale, shock index

## Abstract

**Background:** Blunt liver traumatic injuries are associated with high mortality and remain a significant challenge for modern healthcare systems. The aim of this study was to identify early clinical predictors of mortality in patients undergoing surgery for blunt liver injury. **Material and Methods:** A single-centre, retrospective observational study was conducted involving 27 adult patients who underwent surgery between 2013 and 2025 due to blunt liver injury (traffic accidents, falls from height). Clinical parameters such as GCS and shock index (SI) were correlated with the degree of injury according to the AAST OIS and WSES classifications and with the length of hospitalisation (LOS) using Spearman’s rank correlation coefficient. The study was approved by the Bioethics Committee (resolution number: RNN/86/25/KE). **Results:** Traffic accident victims predominated in the analysed group (81.5%). Deep consciousness disorders (GCS; ρ = −0.65) were shown to be the strongest negative predictor of survival—all deaths occurred in patients with a baseline GCS = 3. A strong positive correlation was also observed between the risk of death and the anatomical level of injury according to the AAST OIS (ρ = 0.70) and shock assessed using the SI (ρ = 0.50). Half of the patients who underwent surgery received packing (tamponade), while 51.8% underwent splenectomy. **Conclusions:** The GCS is the strongest independent clinical predictor of death in this group. Simple neurological and haemodynamic assessment is more useful in practice for quickly identifying critically ill patients than complex anatomical classifications such as AAST or WSES.

## 1. Introduction

### 1.1. Blunt Abdominal Trauma

Traumatic injuries are the primary cause of death in people during the first half of their lives, and the fourth leading cause of death for the entire population. The brain and extremities are most commonly affected, with abdominal injuries being the third most common area of injury (9–15%) [1,2,3]. The vast majority of blunt abdominal injuries are blunt abdominal trauma (BAT), accounting for 80% of all abdominal trauma (AT), while penetrating trauma affects only 20% of patients [4].

Penetrating trauma is commonly caused by knife stabs or gunshot wounds. On the other hand, the most prevalent causes of BAT include traffic accidents (57%), railway accidents (17%), falls from height (15%) and direct blows to the abdomen (6%) [4,5,6]. Nevertheless, it should be emphasised that many sources indicate falls from height as the second most common cause of BAT [7]. Furthermore, people with BAT represent as much as 15% of all victims reporting to A&E due to injuries [8].

BAT is linked to prolonged hospitalisation and a mortality rate of 10–20%, which corresponds to 4.8 million deaths worldwide each year. According to the authors, negative prognostic factors include: severity of injury, extreme age and the presence of multiple organ injuries (polytrauma) [6,7,8].

It should be noted that severe injuries are not only the leading cause of death worldwide, but also pose a significant challenge to healthcare systems and are the primary cause of disability among patients [9].

### 1.2. Liver Trauma

Blunt abdominal trauma (BAT) most frequently results in damage to parenchymal organs such as the liver (38–56%), spleen (33%) and kidneys (23%) [7,10,11,12,13]. Even in 44% of patients admitted to emergency departments with blunt liver injury (BLI), other organs are also affected. Dual organ injuries occur most frequently in the following combinations: liver and spleen (8% of all injuries) and liver and kidneys (7% of all BLI cases) [7].

Among the complications observed in 17% of patients after blunt liver injury (BLI) are: bile leakage, biloma, hepatic or intra-abdominal abscess, haemorrhage, biliary fistula, peritonitis, necrosis, or liver failure. The risk of these complications is closely correlated with the degree of liver injury according to the American Association for the Surgery of Trauma Organ Injury Scale (AAST OIS) [14,15]. The complication rate for AAST grade III is only 1%, for grade IV 21%, while for grade V it is as high as 63% [16].

A connection between mortality and the degree of liver damage according to the AAST OIS classification has also been shown: for grades IV and V, it is 45.9%, while for grades I–III, it is 15.7% [15].

The epidemiology of blunt liver trauma (BLT) mainly affects young adults, whose average age is approximately 30 years, with a clear predominance of males, who constitute over 85% of cases. This demographic structure, as in the case of injuries in general, is explained by men’s greater exposure to risky behaviour [15,17].

It should be pointed out that the majority of liver injuries are minor and usually do not require surgical intervention. Nevertheless, severe liver damage is associated with high morbidity and mortality rates [18,19,20,21,22].

It should be pointed out that the majority of liver injuries are minor and usually do not require surgical intervention. Nevertheless, severe liver damage is associated with high morbidity and mortality rates [18,19,20,21,22]. Although non-operative management has become the preferred approach for hemodynamically stable patients with blunt liver injuries, careful monitoring and appropriate selection remain essential to avoid failure in polytrauma settings [23]. Furthermore, assessing the overall injury burden using established scoring systems is critical for predicting patient prognosis and guiding intervention strategies [24]. Consequently, blunt liver injuries are rarely isolated, and the presence of associated injuries compounding polytrauma significantly escalates the risk of mortality [16,20].

### 1.3. The Role of Modern Imaging Methods and Clinical Experience in the Diagnosis of Injuries

Computed tomography (CT) is the gold standard in diagnosing liver damage resulting from BAT [25]. However, it should be kept in mind that initial CT assessment may underestimate the extent of injury in up to 28% of patients after BAT [26].

In the presence of hemodynamically unstable patients, where the time required to perform a CT scan may delay the implementation of necessary surgical treatment and reduce the chances of survival, it is essential to consider performing an ultrasound examination in the FAST protocol (Focused Assessment with Sonography for Trauma) and eFAST (Extended Assessment with Sonography for Trauma) [27].

An indispensable element of an effective diagnostic process is the involvement of an experienced team, in particular a radiologist, and the availability of specialist facilities within regional trauma centres.

### 1.4. Justification and Purpose of the Work

Current standards of care for liver injuries, based on WSES (World Society of Emergency Surgery) guidelines, largely depend on the patient’s haemodynamic stability and computed tomography (CT) scan, most often categorised according to the AAST-OIS. However, the AAST scale itself, which focuses on the depth of the injury and the size of the haematoma, does not always accurately reflect the complex morphology of the ruptures, which may be crucial for the prognosis. The literature lacks multifaceted analyses that would combine standard assessment (AAST, WSES) with detailed morphological descriptions by Arkuszewski, Conte or Hardy in the context of their impact on survival [16,21,28,29,30].

A key challenge in the Emergency Department (ED) remains the rapid identification of patients for whom non-operative management (NOM) carries a high risk of failure. There is therefore an urgent need to correlate specific patterns of liver damage with readily available clinical parameters such as the Glasgow Coma Scale (GCS), Shock Index (SI), systolic blood pressure (SBP), diastolic blood pressure (DBP), heart rate (HR) and sudden cardiac arrest (SCA).

Our aim is to do a retrospective analysis of clinical data from a leading trauma centre over a 12-year period. The authors attempted to identify an ‘alarm group’—patients with a certain morphological and clinical profile who are at critically high risk of death, which necessitates immediate qualification for surgical treatment, bypassing attempts at conservative therapy.

## 2. Materials and Methods

### 2.1. Research Design

A retrospective, single-centre observational study was conducted involving the analysis of cases of patients hospitalised due to abdominal injuries, in whom further examination confirmed liver rupture as a result of blunt trauma. The analysis covered the period from January 2013 to December 2025. The study was conducted among patients admitted to the Endocrine, General and Oncological Surgery Ward of the M. Kopernik Provincial Multispecialist Centre for Oncology and Traumatology in Łódź (PMCOT).

### 2.2. Eligibility Criteria and Case Identification

Cases were initially identified through a retrospective search of the hospital’s electronic medical records (ClinMed). The base cohort comprised patients diagnosed with abdominal organ injuries, selected using International Classification of Diseases, 10th Revision (ICD-10) diagnostic codes relating to intra-abdominal organ trauma (S36.1—Injury of liver or gallbladder, S36.7—Injury of multiple intra-abdominal organs, S36.8—Injury of other intra-abdominal organs, and S36.9—Injury of unspecified intra-abdominal organ). After this preliminary identification, detailed inclusion and exclusion criteria were applied to determine the final study group.

### 2.3. Inclusion and Exclusion Criteria

A detailed manual analysis of the medical documentation was performed for all eligible patients. The intraoperative assessment of injuries during laparotomy was adopted as the gold standard and indisputable confirmation of liver damage.

Inclusion criteria:

Patients were included in the final analysis if they met all of the following strictly defined criteria:

- Sustained a blunt abdominal trauma exclusively as a result of a road traffic accident (in any mechanism) or a fall from a height.
- Underwent surgical treatment (laparotomy) during which liver injury was indisputably confirmed and assessed intraoperatively.
- Possessed complete medical documentation detailing the circumstances of the injury and the intraoperative morphological description of the liver damage.

Exclusion criteria:

Patients who met the preliminary inclusion criteria were subsequently excluded from the analysis if any of the following applied:

- The patient was managed non-operatively (conservative treatment or interventional radiology only).
- The trauma had a mixed mechanism (coexisting penetrating and blunt trauma).
- Liver injuries of other aetiology, specifically penetrating injuries (e.g., stab wounds or iatrogenic damage).
- The circumstances of the injury were unclear or undocumented, presenting the possibility of spontaneous liver rupture.

Patients with multiple organ injuries, including additional abdominal injuries, were not excluded from the analysis and were assessed on the same basis as other patients.

### 2.4. Identification of Cases

Cases were identified based on a retrospective analysis of electronic medical records available in the internal hospital system (ClinMed). The search was based on ICD-10 codes relating to abdominal organ injuries (S36.0–S36.9). After initial identification, each case was subjected to a detailed manual analysis of the documentation.

### 2.5. Verification and Qualification of Cases

The qualification of cases for the study was conducted independently by two researchers. Each of them independently assessed the medical documentation on the basis of predefined criteria. In the event of discrepancies, a team discussion was held. Disputed cases were ultimately decided by a third researcher with greater experience in traumatology.

### 2.6. Clinical Data and Variables Analysed

Demographic and clinical data were obtained from medical records, including:

- patient’s age and sex;
- mechanism and circumstances of the trauma;
- clinical condition upon admission, including Glasgow Coma Scale (GCS) score, heart rate and blood pressure values;
- clinical symptoms reported upon admission and observed during hospitalisation;
- presence of concomitant injuries.

### 2.7. Diagnostic Imaging

All patients who were able to undergo abdominal computed tomography did so, in accordance with a standard trauma scan protocol. The scans were interpreted by on-call radiologists with experience in trauma diagnosis, working at a regional trauma centre- the M. Kopernik Provincial Multispecialist Centre for Oncology and Traumatology in Łódź (PMCOT).

The descriptive documentation of CT examinations was analysed, with particular emphasis on the diagnosis of liver injury, the nature and location of the damage, and the degree of injury according to the AAST classification, if specified in the radiological or surgical description. The results of imaging examinations were compared with intraoperative findings.

### 2.8. Surgical Treatment

All patients included in the study underwent surgical treatment. Indications for laparotomy were based on clinical presentation, in particular haemodynamic instability and/or signs of internal bleeding. During the procedure, the presence and nature of liver injury, its location and concomitant injuries to abdominal organs were documented.

### 2.9. Injury Severity Scales and Biomechanical Classifications

To provide a standardised assessment of anatomical damages, clinical severity, and the biomechanics of the trauma, all cases were classified using well-established surgical and anatomical scales.

#### 2.9.1. Abbreviated Injury Scale (AIS) and Injury Severity Score (ISS)

The anatomical severity of all sustained injuries was assessed using the Abbreviated Injury Scale (AIS). Each injury was assigned an AIS score ranging from 1 (minor) to 6 (currently untreatable). To quantify the overall trauma severity for each patient, the Injury Severity Score (ISS) was calculated. The ISS is the sum of the squares of the highest AIS scores in the three most severely injured body regions, with possible scores ranging from 1 to 75 [31,32].

#### 2.9.2. American Association for the Surgery of Trauma Organ Injury Scale (AAST OIS)

The degree of liver damage was classified according to the Organ Injury Scale (OIS) developed by the American Association for the Surgery of Trauma (AAST), taking into account the 2018 update [21]. This revision is crucial as it includes vascular criteria (pseudoaneurysms, arteriovenous fistulas) detected by computed tomography (CT) as factors increasing the degree of injury, regardless of the depth of parenchymal rupture.

- Grade I (AAST I): Defined as a subcapsular haematoma involving less than 10% of the surface area or a capsular laceration with less than 1 cm parenchymal depth.
- Grade II (AAST II): These injuries included subcapsular haematomas (<50% of the surface) or parenchymal ruptures <3 cm deep.
- Grade III (AAST III): This includes ruptures >3 cm deep or ruptured subcapsular haematomas.
- Grade IV (AAST IV): Defined as destruction of 25–75% of the liver lobe or active intraparenchymal bleeding.
- Grade V (AAST V): The most severe form of injury, involving destruction of >75% of the lobe or injury to extraperitoneal veins (inferior vena cava, hepatic veins).

#### 2.9.3. World Society of Emergency Surgery (WSES)

In contrast to the AAST OIS, which solely refers to anatomical liver damage, the WSES integrates the anatomical assessment contained in the AAST OIS with the patient’s haemodynamic status [16].

WSES I (Minor): Haemodynamically stable with AAST I-II injuries.
WSES II (Moderate): Haemodynamically stable, but injuries were higher, AAST grade III.
WSES III (Severe): Stable despite extensive liver parenchymal damage (AAST IV-V).
WSES IV (Severe): Haemodynamically unstable patients, which directly reflects the critical condition of victims of traffic accidents and falls from height.

**Figure 1:**
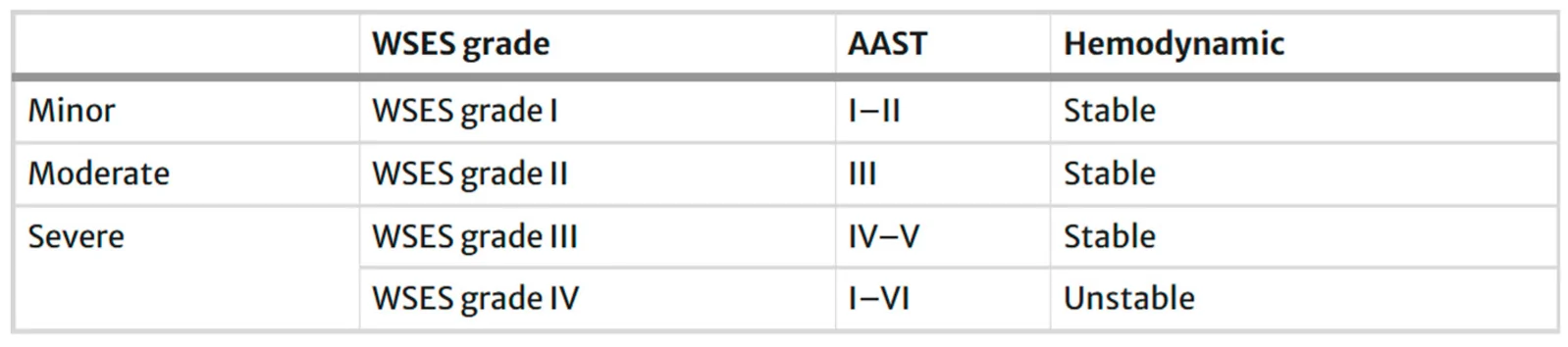
World Society of Emergency Surgery Scale (WSES) [16].

The detailed classification of the WSES is presented in Figure 1.

#### 2.9.4. Arkuszewski’s Classification

The theory of liver deceleration injuries caused by ligaments developed by Arkuszewski is based on a biomechanical analysis of blunt liver trauma, with particular emphasis on the role of deceleration and shearing forces acting on the vulnerable liver parenchyma through its own ligamentous apparatus [28].

- Type A1: The rupture occurred along the right triangular ligament, which is strongly associated with horizontal deceleration (particularly in the case of traffic accidents).
- Type A2: The rupture of the left lobe along the falciform ligament as a result of multi-vector deceleration.
- Type A3: As in type 2, there was a rupture of the left lobe, but the cause was shear forces associated with pulling on the coronal or left triangular ligament.

A detailed description of the classification proposed by Arkuszewski et al. is presented in Table 1.

#### 2.9.5. Conte Model

A complete Conte classification of injury patterns, depending on the mechanism of compression and crushing [29]:

Type 1: Laceration of the diaphragmatic face of the right lobe, adjacent to the bare area.
Type 2: Laceration of the right anterior lateral face with extension to the posterior face.
Type 3: Laceration of the lower part of the right lobe affecting more than half of the lobe.
Type 4: Laceration initiated next to the vena cava.

#### 2.9.6. Hardy’s Model

The historical classification, which forms the basis of contemporary biomechanical divisions [30].

Type 1 Hardy: Laceration of the liver parenchyma, most commonly located in the posterior-superior segment of the right lobe.

Type 2 Hardy: Large, usually multiple and deep ruptures, running vertically or obliquely downwards and forwards. These injuries closely correlate with the modern Type 2 according to Arkuszewski, reflecting tears running along the falciform ligament.

### 2.10. Statistical Analysis

Statistical analysis was performed using Python version 3.14 with the IDLE environment (Python Software Foundation, Wilmington, DE, USA). The statistical analysis was descriptive. Data were presented using means and standard deviations for continuous variables and frequencies and percentages for qualitative variables. Due to the small size and heterogeneity of the study group, no formal meta-analysis or comparative tests were performed. The analysis was limited to a narrative description and simple statistical analysis.

### 2.11. Ethical Aspects

The study received a favourable opinion from the relevant bioethics committee (resolution number: RNN/86/25/KE). The committee decided that the study did not need individual consent from patients because it was retrospective, only used medical records, and did not interfere with treatment or involve direct contact with patients. All data were fully anonymised before analysis.

### 2.12. STROBE Standards

The study was conducted in accordance with STROBE guidelines [33].

## 3. Results

### 3.1. Study Population

**Figure 2:**
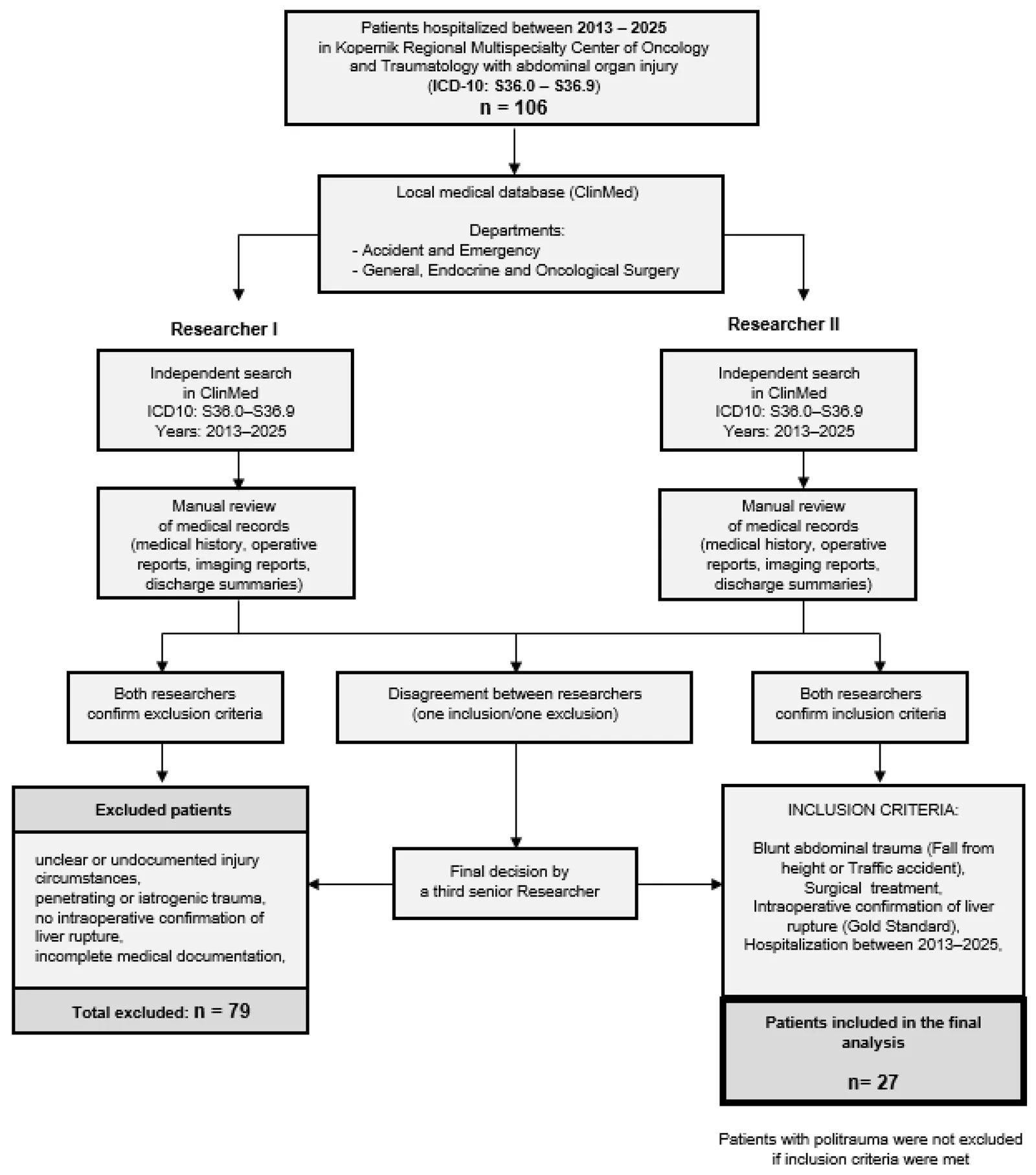
Process of identifying, verifying and qualifying patients for the study.

During the analysed period, 106 patients hospitalised with a diagnosis of abdominal organ injury, coded according to the ICD-10 classification (S36.0–S36.9), were identified in the hospital’s electronic database. After preliminary analysis of medical records and application of inclusion and exclusion criteria, 27 patients with confirmed liver injury due to blunt trauma (falls from height or road traffic accidents) were selected for further evaluation. All patients underwent surgery, and the diagnosis of injury was confirmed intraoperatively. A detailed diagram of the patient identification and qualification process is presented in Figure 2. Ultimately, these 27 patients were included in the statistical analysis, constituting the study population.

#### 3.1.1. Study Identification and Selection of Patients

The data were extracted from the internal hospital information system AMMS, which is routinely used by medical staff at the M. Kopernik Provincial Multispecialist Centre for Oncology and Traumatology in Łódź. (Poland) for patient data collection and medical record management. A detailed annual distribution of both the initially identified cohort (by ICD-10 codes) and the final eligible study population (by injury mechanism) is presented in Table 2.

Between 2013 and September 2025, a total of 106 cases were initially identified. Among them, 4 were duplicates caused by overlap between the primary category (S36.1—injury of liver and gallbladder) and the subcategories (S36.7–S36.9). Such overlaps are indicated in parentheses. After a detailed review of medical records and application of inclusion and exclusion criteria, 27 unique cases of liver injury associated with traffic accidents or falls from height remained eligible for final analysis.

The number of cases varied across years. The highest count was noted in 2023 (16 cases, including 2 duplicates), while the lowest was observed in 2013 and 2018 (2 and 3 cases, respectively). Importantly, the year 2025 already shows nearly the same number of cases (15) as the peak year 2023, despite covering only the period up to September, which may suggest a continuing upward trend in incidence. Overall, the data indicate a gradual increase in recent years, which could reflect both a true rise in the occurrence of such injuries as well as improved coding and reporting practices. It is only since 2025 that the Clinic has been providing an emergency service every day; previously, the number of services per month was lower and varied. This fact may have influenced the distribution of the number of cases across individual years. These preliminary findings provide an overview of the frequency of ICD-10-coded liver injuries over the analysed period. They also highlight the need for adequate planning of trauma and surgical care resources, given the potential rise in the number of such cases in the near future.

The most common reasons for excluding cases from the final analysis included liver injuries resulting from penetrating trauma (stab wounds) rather than blunt abdominal trauma. In addition, cases without clear intraoperative confirmation of liver injury were excluded, as well as those managed conservatively without laparotomy. Finally, injuries associated with falls from standing height were also excluded, as they did not meet the predefined inclusion criteria.

After a detailed review of medical records, including laboratory tests, imaging studies, and operative reports, 27 unique cases of liver injury associated with traffic accidents or falls from height remained eligible for final analysis. Out of these 27 cases, the majority—22 cases (81.5%)—resulted from traffic accidents, while 5 cases (18.5%) were caused by falls from height. This dataset allows insight into the relative frequency of different trauma mechanisms among patients with confirmed liver injuries and can guide further clinical or epidemiological analysis.

#### 3.1.2. Demographic Characteristics

This study involved 27 cases of patients who suffered liver injuries as a consequence of blunt abdominal trauma. Among the patients, there were 16 males (59.3%) and 11 females (40.7%) aged between 19 and 59 years (mean age 35.9 ± 12.4).

In total, 22 of them experienced abdominal trauma as a direct result of a traffic accident, including 12 men (54.5% of traffic accidents) and 10 women (45.5%). The age range of this group of patients was consistent with the age range of the entire study population. Of this population, six people (27.3%) were drivers, six people (27.3%) were passengers, five (22.7%) were pedestrians, one person (4.5%) was a motorcyclist, and the detailed circumstances of four patients’ injuries are unknown, but their nature clearly indicates a traffic-related cause.

The remaining five patients admitted to the Department suffered injuries due to falls from height. This subgroup consisted of four men (80%) and one woman (20%). The average age of patients in this subgroup was 40.2 years (±11.8), and the age range was between 20 and 49 years.

The detailed demographic breakdown by subgroup is summarised in Table 3.

#### 3.1.3. Clinical Condition on Admission and Initial Assessment of Shock

The following parameters were analysed upon admission to the Department of Emergency Medicine: haemodynamic analysis (HR, SBP, SI) and consciousness assessment (GCS).

In our study, the average Glasgow Coma Scale (GCS) score was 9.7 ± 5.4, with a full range and a median of 10. The average systolic blood pressure (SBP) was 106 ± 38 mmHg (range 0–165 mmHg, median 102 mmHg), while the mean heart rate (HR) was 98 ± 34 beats per minute (bpm) (range 0–140, median 100). In 5/27 cases, sudden cardiac arrest occurred. The Allgower Index (Shock Index—SI) was 0.89 ± 0.58, with a wide range of values–from haemodynamic stability to life-threatening shock (0.02–2.33) and a median of 0.9, which highlights the significant haemodynamic instability in the study population.

In a subgroup of patients who had been involved in traffic accidents (*n* = 22), the mean GCS score was slightly lower at 8.5 ± 5.5 (range 3–15, median 7.5), indicating a higher incidence of moderate to severe consciousness disorders in this group. The average SBP was 98 ± 34 mmHg (range 0–156, median 100), while the average HR was 103 ± 33 bpm (range 0–140, median 100). SI was elevated compared to the general population and averaged 1.13 ± 0.44 (range 0.57–2.33, median 1.1), which is equivalent to a greater severity of circulatory disorders in patients after traffic accidents. In this group, two cases (9.1%) of sudden cardiac arrest were reported.

Conversely, in the subgroup of patients who had fallen from a height (*n* = 5), the lowest mean GCS scores were recorded, at 6.0 ± 4.2 (wide range 3–14, median 4), suggesting significantly more severe disturbances of consciousness. The mean SBP was similar to that observed in other groups and was 102 ± 58 mmHg (range 0–165, median 124) with a lower mean heart rate (75 ± 43 bpm, range 0–130, median 80). The shock index in this group was slightly lower than in patients after traffic accidents and averaged 0.84 ± 0.46 (range 0.48–1.63, median 0.63). In this group, more than half of the patients (3/5, 60%) suffered sudden cardiac arrest.

All patients who died had a GCS score of 3 on admission to hospital. Among those who survived, however, GCS scores ranged from 4 to 15. The mean GCS score for road traffic accident victims was 8.5, which was higher than that for victims of falls from a height, where it was 6.0.

The shock index for road traffic accident victims was 1.13, which was higher than that for victims of falls from a height, where it stood at 0.84.

Table 4 shows a summary of key physiological parameters and Allgower’s index (SI) values in the entire study population and in individual injury subgroups.

#### 3.1.4. Injury Categorization—Volumetric Assessment of Parenchyma (AAST OIS)

Analysis of the material showed that the study population was characterised by an overrepresentation of severe liver injuries, which is typical for a referral trauma centre.

- Grade II (AAST II): 5 patients (18.5%). All patients in this group survived.
- Grade III (AAST III): 9 patients (33.3%). The mortality rate in this group was 22.2% (2/9), with deaths (e.g., S.K., A.K.) possibly resulting from accompanying injuries.
- Grade IV (AAST IV): 8 patients (29.6%). There were 2 deaths in this group (25% mortality).
- Grade V (AAST V): 5 patients (18.5%). The mortality rate in this group was catastrophic, at 80% (4/5).

#### 3.1.5. Injury Categorization—Patient Physiological Status (WSES)

The distribution in the study group (N = 27) confirms the very severe nature of the events:

- WSES I (Minor): 3/27 patients (11.1%).
- WSES II (Moderate): 2/27 (7.4%).
- WSES III (Severe) 6/27 (22.2%).
- WSES IV (Severe): 16/27 (59.3%). This is the most numerous group.

#### 3.1.6. Injury Categorization—Sudden Deceleration Mechanism (Arkuszewski Model)

The above classification was applicable in 13/27 patients (48.1%). Of these:

- Type A1: 4/27 patients (14.8%) were classified.
- Type A2: 4/27 patients (14.8%, including 1 mixed case).
- Type A3: 6/27 (22.2%, including 1 mixed case).

#### 3.1.7. Injury Categorization—Crushing Mechanism (Conte Model)

No pure Type 1 or Type 5 cases were found in the analysed group. The rest are described below:

- Type 2: 4/27 patients (14.8%);
- Type 3: 4/27 patients (14.8%);
- Type 4: 3/27 patients (11.1%).

#### 3.1.8. Injury Categorization—Historical Reference Pattern (Hardy’s Model)

In total, 7 patients (7/27; 25.9%) were classified. All eligible patients were classified as type 2 according to Hardy’s model.

#### 3.1.9. Injury Categorization—Injury Severity Score (ISS)

Analysis of the data demonstrated that the study population was characterised by an exceptionally high severity of polytrauma injuries. All patients had an ISS exceeding 15 points (classified as major trauma). The distribution in the study group (N = 27) is as follows:

- ISS < 25 (severe injuries): 4 patients (14.8%). All patients in this group survived (mortality rate 0%).
- ISS 25–49 (very severe injuries): 13 patients (48.1%). Two deaths were recorded in this group, resulting in a mortality rate of 15.4% (2/13).
- ISS ≥ 50 (critical injuries): 10 patients (37.0%). The mortality rate in this subgroup was extremely high, at 70.0% (7 out of 10 patients). The vast majority of all deaths in the study population occurred in this group.

#### 3.1.10. Categorisation of Injuries—Abbreviated Injury Scale (AIS)

Due to the inclusion criteria, all patients (27/27; 100%) had abdominal injuries (AIS4) with a severity score of ≥3, predominantly grade 4 and 5 injuries (11 and 10 patients, respectively). An analysis of associated injuries, classified as severe or critical (AIS score ≥3 in a given region), revealed the following distribution:

- Thoracic region (AIS3 ≥ 3): 21 patients (77.8%). This was the most common site of severe associated injuries (including multiple rib fractures, pulmonary contusions and pneumothorax).
- Limbs and pelvis (AIS5 ≥ 3): 19 patients (70.4%). This group was dominated by complex fractures of the long bones and pelvic instability.
- Head and neck (AIS1 ≥ 3): 17 patients (63.0%). This group included patients with severe traumatic brain injuries (TBI), including two extremely critical cases assessed as AIS 5 (patients 14 and 27).
- Craniofacial (AIS2 ≥ 3): 1 patient (3.7%).
- Skin and soft tissues (AIS6 ≥ 3): No severe injuries in this area were recorded in the study group (all AIS6 cases were assessed as 1 or 2).

#### 3.1.11. Injury Categorization—Integrated Trauma Patient Profile

The summary of the study group shows that the extreme energy of road traffic accidents and falls from height dominates in the material and directly translates into the highest clinical severity levels (AAST OIS IV-V, WSES III-IV). This profound trauma burden is further demonstrated by the exceptionally high Injury Severity Scores (ISS) and extensive concomitant injuries across multiple body regions (assessed via the Abbreviated Injury Scale, AIS). A parallel assessment of biomechanics reveals the significant role of deceleration, which leads to specific damage to the ligamentous apparatus, regardless of compression itself. Nevertheless, it should be emphasised that the retrospective nature of the study and the associated lack of precise morphological data in the documentation of patients in the most severe condition prevented a complete biomechanical classification in several cases.

A detailed classification and characteristics of liver injuries are summarised in Table 5. Classifications based on both deceleration (Arkuszewski) and compression (Conte) mechanisms are included, taking into account historical background (Hardy) and correlation with the modern WSES/AAST clinical scale [16,21,28,29,30]. Additionally, the comprehensive individual trauma profiles, detailing regional AIS scores, total ISS values, and specific associated injuries, are presented in Table 6.

Meanwhile, detailed data on the prevalence and types of associated injuries identified, broken down by anatomical region, are presented in Table 7.

### 3.2. CT Findings vs. Intraoperative Findings

#### 3.2.1. CT-Based Diagnosis of Liver Injury

In our study group of 27 patients with liver injury, preoperative computed tomography (CT), which is the basis for radiological assessment, was performed in 17 patients (63.0%). Due to haemodynamic instability and the need for emergency surgery, early imaging diagnostics were not performed in 5 patients (18.5%). In another 5 patients (18.5%), CT was performed only in the postoperative period or medical data in this regard was missing.

#### 3.2.2. Verification of Individual Radiological Diagnoses

Analysis of the concordance between radiological diagnoses and intraoperative verification demonstrated that CT reliably identified unambiguous ruptures—all 6 described ruptures were confirmed intraoperatively (6/6, 100%). Simultaneously, however, CT scans often failed to detect liver ruptures and instead classified them as less serious lesions: of the 11 descriptions as contusions (1 case without verification), only 2 of the 10 verifiable cases turned out to be factual contusions (2/10, 20%), while in 8/10 cases (80%) CT underestimated the injuries—parenchymal ruptures were found during surgery. All four descriptions of ‘no changes’ proved to be false negatives (0/4, 0%) and corresponded to serious damage (ruptures, lacerations, dissection), and both descriptions of haematoma were not confirmed intraoperatively (0/2, 0%)—in fact, active liver ruptures were found. In turn, the categories suggesting more severe damage, i.e., suspected rupture and laceration/fissure, showed full compliance with verification (2/2, 100% each), while the only case marked as suspected haematoma turned out to be a rupture (0/1, 0%). Three descriptions classified as post-traumatic changes were non-specific and did not allow for an unambiguous assessment of the presence of a rupture. The precise characteristics and classification of the above variables for radiological diagnosis are presented in Table 8.

#### 3.2.3. Assessment of Diagnostic Accuracy and Injury Underreporting Profile

To assess diagnostic accuracy, a subgroup of 14 patients was selected in whom it was possible to compare the results of preoperative CT scans with the actual findings in the operating theatre. Full concordance, in which the imaging test accurately and precisely described the liver rupture, was noted in 2 patients (14.3% of the assessed subgroup). Partial agreement, consisting of an accurate suspicion of injury without certainty of diagnosis, was found in 3 patients (21.4%). In most cases, i.e., in 9 patients (64.3%), the CT scan drastically underestimated the actual degree of organ damage.

A careful analysis of 9 underestimated cases suggested that the most common discrepancy (5 patients, 55.6% of misjudgements) was diagnosing a milder parenchymal contusion on CT, which turned out to be a severe rupture during surgery. In 2 cases (22.2%), imaging indicated the presence of a haematoma (post-traumatic changes were described instead of active structural damage). In the remaining 2 cases (22.2%), CT completely missed the injury—although the description indicated no changes, liver ruptures were found during surgical verification. The complete features of the imaging analysis are presented in Table 9.

### 3.3. Statistical Analysis of Predictive Variables

Due to the lack of normality of the distribution of all key variables, confirmed by the Shapiro–Wilk test (none of the analysed variables were consistent with the Gaussian distribution), common parametric tests (Student’s *t*-test, Pearson’s correlation) were not applied. Therefore, Spearman’s non-parametric rank correlation, which does not require the assumption of normal data distribution, was used for further analysis. Furthermore, it is more tolerant of the presence of outliers. The choice of this method was especially justified because of the small sample size and the clinically heterogeneous nature of the study population.

The aim of the statistical analysis was to determine the strength and direction of the correlation between selected clinical variables and the mortality risk in patients with blunt abdominal trauma with liver injury. The results of Spearman’s correlation are presented in Table 10. All obtained correlation coefficients (ρ) were statistically significant (*p* < 0.001). The strongest positive correlation with the risk of death was observed for the degree of liver damage assessed in AAST OIS (ρ = 0.70), which implies that as the severity of organ injury increases, the risk of death increases significantly. An equally significant, albeit negative, correlation was found between the mortality risk and GCS (ρ = −0.65), showing that deeper disturbances of consciousness are associated with a worse prognosis.

A moderately positive correlation with the risk of death was also found for SI (ρ = 0.5), confirming the importance of haemodynamic instability as a prognostic factor (albeit with less predictive power than AAST OIS). The strongest negative correlation with the risk of death was observed for length of hospitalisation (LOS. ρ =−0.74). This indicates that deaths occur significantly more often in the initial period of treatment, while survival is associated with a longer hospital stay. Additionally, an equally strong negative correlation (ρ = −0.74) was shown between AAST OIS and GCS, suggesting that severe liver injuries often coexist with profound disturbances of consciousness (often in the course of multiple organ injury or haemorrhagic shock).

**Figure 3:**
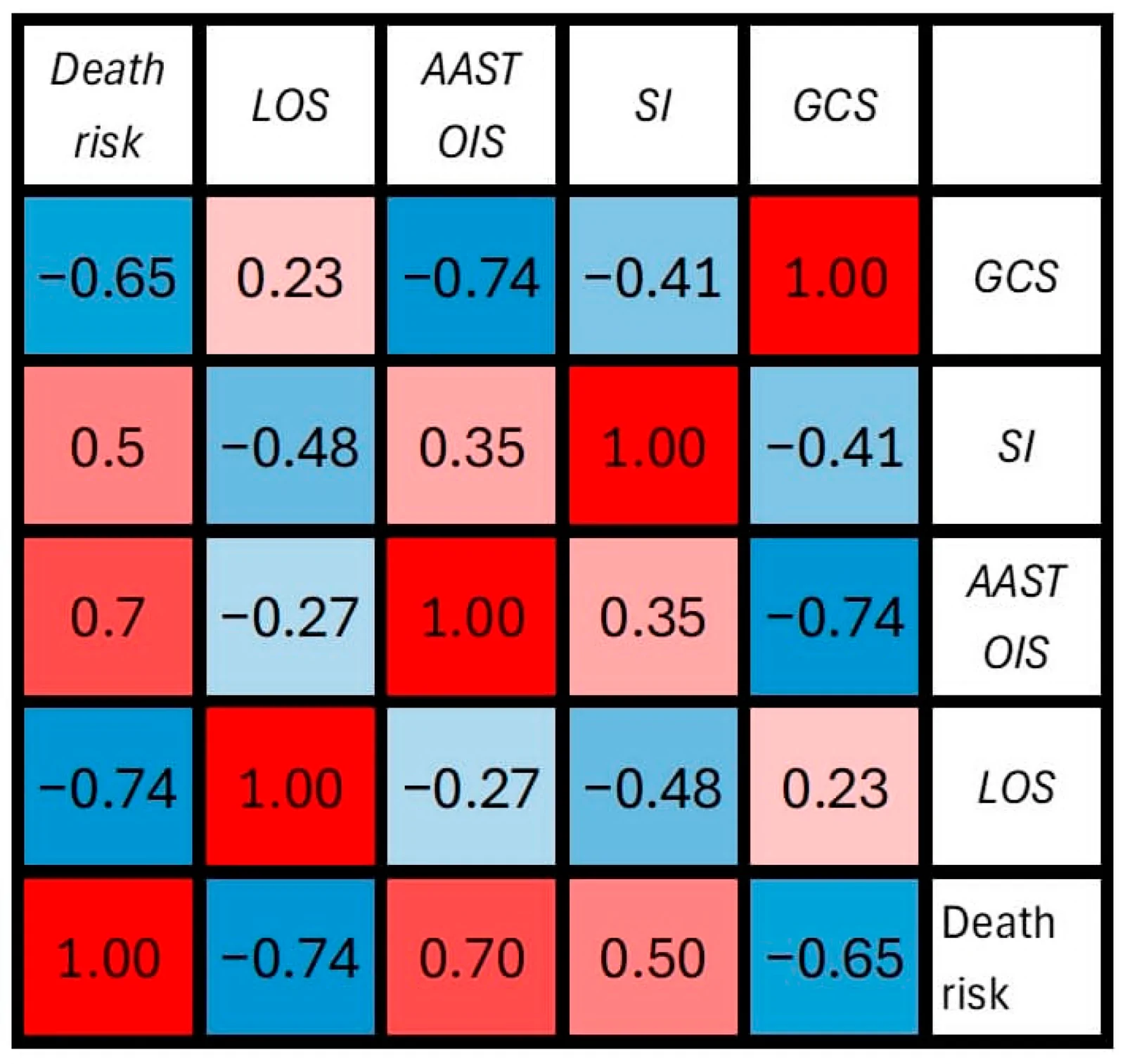
Spearman’s correlations between clinical parameters and risk of death in patients with blunt liver injury—heatmap. GCS—Glasgow Coma Scale; SI—Shock Index (SI = HR/SBP); LOS—Length of Stay; AAST OIS—American Association for the Surgery of Trauma Organ Injury Scale.

The aforementioned results also appear in the form of a heat map (Figure 3), which enables a synthetic and visual assessment of the mutual connections between the analysed variables.

**Figure 4:**
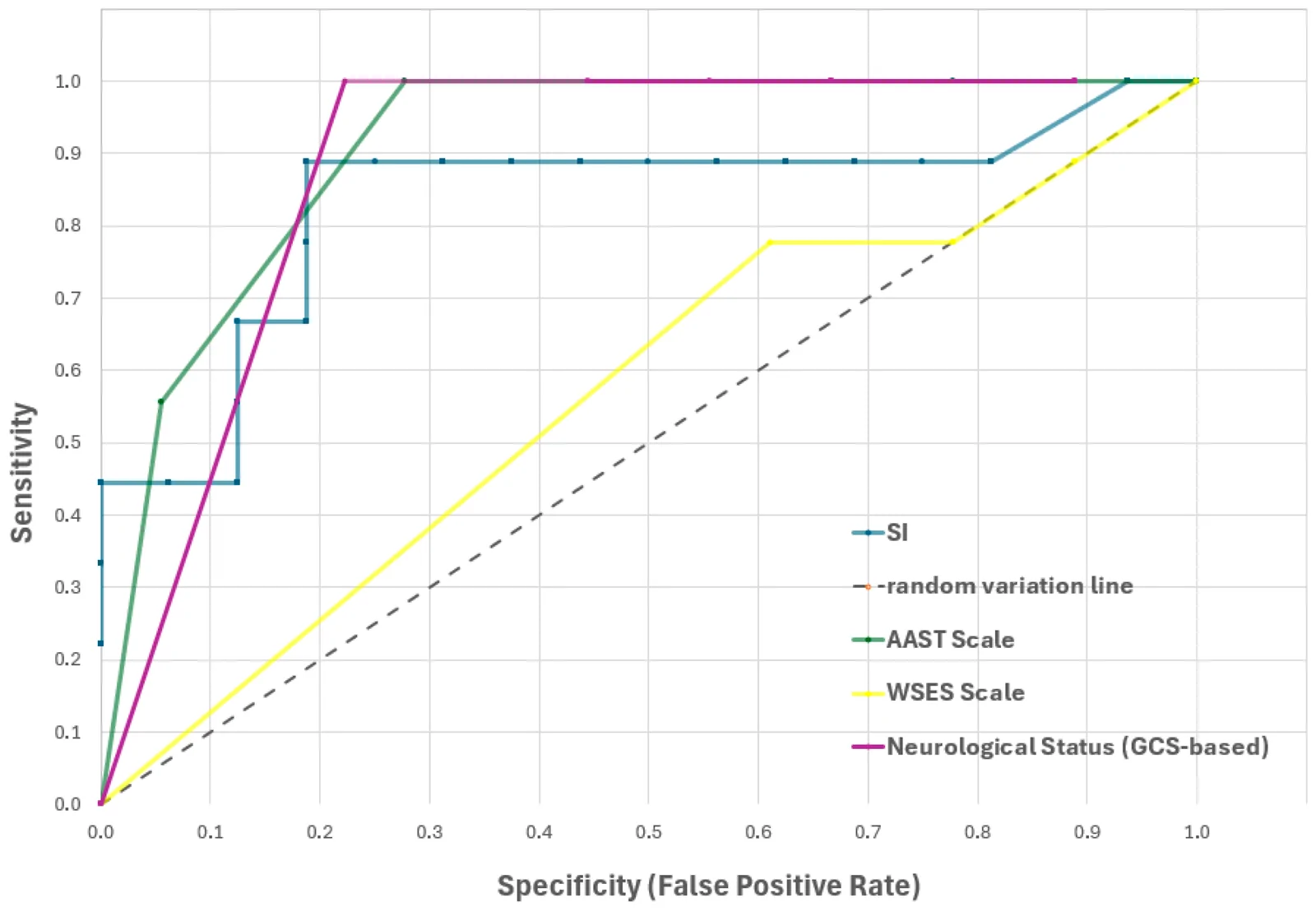
ROC curve for the selected predictive factors.

**Figure 5:**
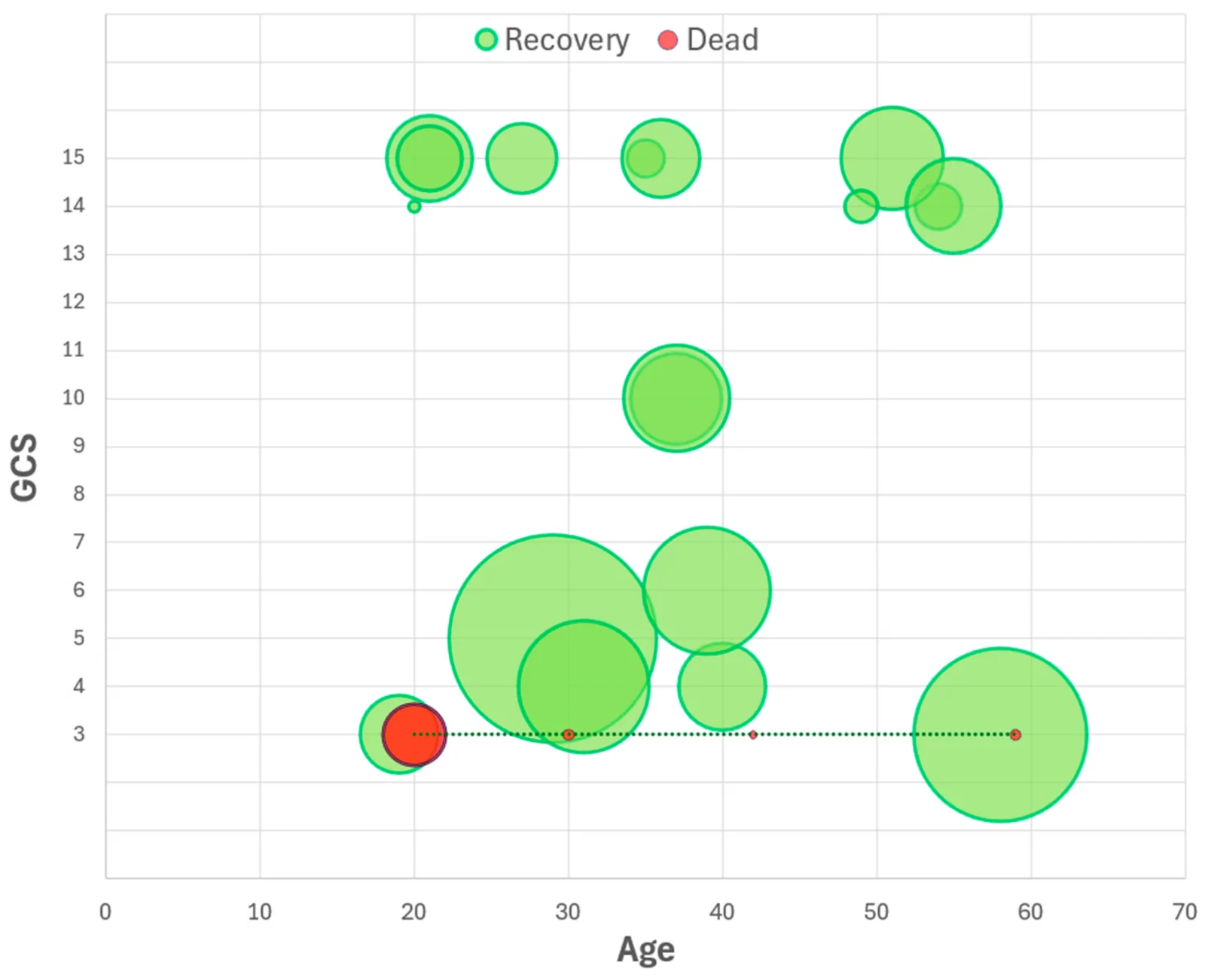
Multidimensional analysis of patient distribution: relationship between age and GCS score and length of stay (LOS). The critical impact of severe unconsciousness is noticeable—all deaths in the study group (red dots connected by a line) occurred exclusively in patients with a minimum GCS score of 3, regardless of age. Simultaneously, the largest green circles at the bottom of the graph indicate that patients with low baseline GCS who survived the injury required the longest hospital stay.

ROC chart showing the predictive value of clinical parameters in liver traumatic injuries. Neurological status (consciousness disorders) is the earliest and strongest warning sign, surpassing haemodynamic parameters in terms of stability. In the study group, where high-grade injuries (IV-V according to AAST) were associated with almost 100% mortality, the commonly recommended WSES showed less usefulness and sensitivity than the isolated AAST or GCSs. The detailed data are presented in the Figure 4 and Figure 5, on the contrary, illustrates the relationship between age, GCS score and length of stay (LOS).

### 3.4. Laparotomy and Packing

Laparotomy was performed in all 27 patients, confirming the aggressive surgical approach in this cohort.

Packing: Perihepatic packing was reported in 11 patients (40.7%). This is a key element of the Damage Control Surgery (DCS) strategy, used in patients with coagulopathy, acidosis and hypothermia (the ‘triad of death’) in whom definitive surgical treatment is impossible or too risky.

Packing intensity: The number of gauze pads used ranged from 1 to 14 (mean: 6.3). The record number (14 gauze pads) was used in patient D.N. (AAST V, death), which indicates a desperate attempt to control massive haemorrhage.

Packing-AAST correlation: The use of packing was strongly associated with a higher AAST grade. In the AAST V group, packing was used in 100% of operated patients.

In the remainder of the patients, in addition to suturing the liver rupture, peritoneal lavage and drainage were performed in 8 patients (29.6%), a haemostatic dressing (Tachocomb or Spongostan) in 5 patients (18.7%), and argon coagulation of smaller ruptures combined with lavage in 3 patients (11.1%).

### 3.5. Splenectomy—Accompanying Procedure

Splenectomy was performed in 14 out of 27 patients (51.8%). In the Falls group: Splenectomy was necessary in as many as 4 out of 5 patients (80%). In the Accidents group: Splenectomy was performed in 10 out of 22 patients (45.5%).

## 4. Discussion

### 4.1. Epidemiology and Trend

The authors want to emphasise the important distinction between the terms trauma and injury, which are sometimes used interchangeably in the literature [34], but in the context of this paper they have different meanings. The term trauma refers to the action of an external factor (e.g., a punch, a kick, a fall) that initiates a series of pathophysiological changes in the body, while injury is interpreted as the specific tissue or organ damage resulting from this event. It should be emphasised that the limited size of the study group necessitates cautious interpretation of our conclusions. Nevertheless, the observations obtained are of such significant value for clinical practice that we have decided to present them in detail.

The demographic data collected indicate a clear upward trend in the frequency of liver injuries diagnosed at our regional trauma centre. This phenomenon is most likely not due to an increase in the absolute number of traffic accidents or falls from height. The authors suggest that more accurate coding and reporting of medical cases, as well as the development of emergency medical algorithms, play a key role here. The increased availability of emergency medical teams, faster transport of patients in serious condition to the destination trauma centre, more effective pre-hospital interventions and the more widespread use of modern imaging methods directly translate into higher detection rates for this type of injury.

### 4.2. Clinical Condition

Due to the specific nature of the facility where the study was conducted, which serves as a regional trauma centre, severe liver injuries (grades III-V on the AAST scale) predominated in the analysed population, which is typical for this type of referral centre. It is worth noting that grade V injuries on the AAST scale were associated with a high mortality rate of up to 80%.

Despite the blunt nature of the injury mechanism, a decision was made to divide the study population into two subgroups: patients after traffic accidents and patients after falls from height. This division was dictated by the different leading clinical problems in both groups. Patients after traffic accidents (*n* = 22) had a higher shock index (Allgöwer index), suggesting that the main threat in this group was severe haemodynamic disturbances resulting from haemorrhage from a damaged liver. In contrast, patients after falls from height had significantly lower GCS scores (mean 6 points). This result indicates that severe craniocerebral injuries were the dominant component in this subgroup. Furthermore, more than half of the patients (3/5) in this group suffered sudden cardiac arrest (SCA). The above data suggest that the circumstances and mechanism of injury determine the leading clinical problem: haemorrhagic shock of abdominal origin as opposed to polytrauma with predominant central nervous system injury.

This clinical dichotomy is clearly reflected in our results (Table 7), which indicate a high prevalence of multi-organ injuries—particularly concomitant chest and head injuries accompanying abdominal organ injuries. This anatomical distribution emphasises that blunt liver injury in a trauma centre setting rarely occurs in isolation, which supports the concept that patient outcomes depend largely on the combined burden caused by severe multi-organ injury, rather than on the failure of a single organ.

Nevertheless, it is important to emphasise the unique role of the liver, given its complex vascular structure and its proximity to the major blood vessels. For this reason, severe liver injury is inherently associated with a high risk of life-threatening haemorrhage, making it one of the leading causes of mortality in patients with severe injuries [35].

### 4.3. Trauma Biomechanics

The analysis showed that in 48.1% (13/27) of patients, liver injuries could be attributed to a deceleration mechanism. These were, in fact, tears located along the hepatic ligaments. According to the concept presented by Arkuszewski et al. [28], parenchymal tears are often caused by shear forces generated by the sudden movement of the organ relative to the ligamentous apparatus: the right triangular ligament (type A1) or the falciform and coronary ligaments (types A2 and A3). These observations prove that liver rupture occurs not only as a result of direct crushing under compressive forces. At the moment of sudden deceleration, as occurs, for example, during a head-on collision, the shearing forces generated act on the ligamentous apparatus, which directly leads to tearing of the liver parenchyma, even in the absence of local signs of direct trauma to the abdominal wall [36,37].

### 4.4. CT and Diagnostic Traps

An extremely important observation in the study group is the fact that in as many as 64.3% (9/14) of verifiable cases, computed tomography (CT) underestimated the actual extent of damage to the liver capsule and parenchyma. The most common discrepancy (5/9 incorrect assessments) was the diagnosis of a mild contusion in the imaging examination, which during surgical intervention turned out to be a severe organ rupture. A similar phenomenon of masking the actual clinical condition was also observed in cases originally described as haematoma or no pathological changes. This suggests that contusions described by radiologists in the acute phase of injury often mask much deeper parenchymal tears [38]. This phenomenon may result from the fact that fresh blood, damaged tissue and oedema coexisting with the acute phase of shock and accompanying vascular constriction present a density similar to that of a rupture in CT scans, leading to the merging of these structures in the radiological image. These data carry a fundamental message for clinical practice: the surgical team should never base their sense of security solely on the results of imaging tests. In the case of a patient with blunt liver injury, especially a haemodynamically unstable patient, the sudden need for exploratory laparotomy should always be taken into account.

### 4.5. Predictors of Mortality

The WSES (World Society of Emergency Surgery) scale is widely recommended as a universal classification tool for multiple trauma [16]. It is worth noting that this scale integrates the anatomical degree of organ damage with the patient’s current haemodynamic status, making it seem like an ideal tool for assessing the study group. Nevertheless, our study showed that this scale could underestimate the critical risk in some patients, especially in the group with predominant severe craniocerebral trauma. With regard to the entire study group, the patient’s neurological status classified on the GCS, as well as the individual assessment of the anatomy of injuries according to the AAST OIS, proved to be a much stronger and earlier predictor of early death. It is worth noting that AAST IV and V injuries were associated with the highest mortality rates. The above observations suggest that the WSES should be used with caution as the sole predictor of mortality in patients with complex polytrauma, where the synergistic effect of multiple concurrent injuries (such as severe traumatic brain injury combined with major hepatic haemorrhage) frequently overrides anatomically based scoring systems and dictates early mortality risk [16].

Moreover, the clinical presentation and prognosis in patients with blunt liver trauma cannot be considered solely in terms of abdominal pathology. As shown by data from our centre (Table 6 and Table 7), every patient had associated injuries, and there were no cases of isolated liver trauma. The high prevalence of concomitant chest injuries -such as multiple rib fractures and the pneumothorax that frequently accompanies them-combined with severe fractures of the pelvis and limbs, as well as intracranial injury (e.g., subarachnoid haemorrhage and traumatic brain injury), places an additional pathophysiological burden on the patient. For example, an analysis of individual patient profiles (Table 6) reveals that cases resulting in early death (such as cases 5, 6 and 14) were often characterised by high AIS scores in multiple body regions simultaneously (e.g., AIS 4–5 in both the thorax/abdomen and the cranium/limbs) which resulted in the Injury Severity Score (ISS) significantly exceeding 50 points.

This multi-organ injury means that life-threatening systemic consequences such as acute respiratory failure caused by pulmonary contusions, severe coagulopathy and secondary brain injury increase the risk of mortality. Consequently, the above findings suggest that the WSES should be used with caution as the sole prognostic factor for mortality in patients with complex multi-organ injury, in whom the concurrent deterioration of extrahepatic organ systems often takes precedence over scoring systems based solely on the liver or purely anatomical criteria, and determines the ultimate survival outcome.

### 4.6. Damage Control Surgery

A modern and minimally invasive method for the treatment of blunt liver trauma is transarterial angioembolisation [39]. It is a highly effective method for controlling arterial haemorrhage from the liver, is well tolerated and has a low complication rate [14,40]. However, angiographic embolisation has limited applicability in patients with polytrauma and haemodynamic instability of multifactorial aetiology, whose critical condition requires immediate surgical intervention instead of endovascular procedures. None of our patients underwent angiembolisation following emergency laparotomy. This was because the haemodynamic status of some patients had stabilised, whilst the others died soon after the operation.

The results of our study clearly confirm that damage control surgery (DCS) is the gold standard in the surgical treatment of blunt liver injuries in patients with multiple organ injuries and severe haemodynamic instability [27]. The use of techniques such as abdominal packing (used in 50% of operated patients) enables rapid and effective control of massive haemorrhage. This procedure interrupts the deadly cascade of pathophysiological changes (the so-called triad of death), creating a therapeutic window necessary to improve the patient’s haemodynamic status in the intensive care unit and ultimately reduce the early risk of death [41]. The use of packing in patients with AAST V was appropriate given the nature of this degree of liver damage.

### 4.7. Limitations of the Study

This study has certain limitations that ought to be taken into account when interpreting the results. First of all, the retrospective nature of the analysis and its limitation to a single reference centre (single-centre study) may affect the representativeness of the sample and limit the possibility of directly extrapolating the conclusions to the overall population. It is only since 2025 that the Clinic has been providing an emergency service every day; previously, the number of services per month was lower and varied. This fact may have influenced the distribution of the number of cases across individual years.

An important methodological factor was that the analysis was based on computed tomography (CT) scan descriptions and intraoperative diagnoses. Although this approach accurately reflects the daily clinical reality of working in an emergency department, it may involve some subjectivity in assessment and inter-observer variability.

Another limitation is the size of the study group and the heterogeneity of injury mechanisms, which prevented the use of more advanced statistical models or the performance of a formal meta-analysis. It should also be noted that there is a lack of data on long-term follow-up after patient discharge from hospital.

Last but not least, a limitation of this study is the multifactorial character of the observed decline in GCS scores. Although liver injury and secondary haemorrhagic shock were common to the entire study group, in some patients the reduction in level of consciousness may have been due to accompanying traumatic head injury. This additional impact of traumatic head injuries represents an important confounding factor that must be taken into account in the final interpretation of the results.

Nevertheless, despite these limitations, the study provides important data of practical value. Focusing on the actual decision-making process allows for a reliable comparison of the diagnostic effectiveness of CT with intraoperative verification (laparotomy), which is crucial for optimising the management of trauma patients in life-threatening conditions.

## 5. Conclusions

1. The strongest independent predictor of death in the study group was the Glasgow Coma Scale (GCS), followed by the shock index (SI), confirming the paramount role of neurological and haemodynamic assessment in blunt liver injury. However, the predictive value of the Glasgow Coma Scale is not significantly useful, as all patients with a GCS score of 3 died. By contrast, the lowest score recorded in a patient who survived was GCS = 4.
2. Complex classification systems, including the WSES and the AAST anatomical scale, have proven to be less useful in identifying critical patients than a simple assessment of neurological and haemodynamic status.
3. Well-established classifications of liver injuries are validated in clinical practice, although they have no prognostic value.
4. In cases of liver injury following blunt trauma, the mechanism by which the injury occurs is complex, and both compression of the abdominal cavity and deceleration and shear forces may play a role.

## Figures and Tables

**Table 1:** Detailed description of Arkuszewski’s classification.

Description	Lacerations Near and Along the Liver Ligaments—Type A	Lacerations as in Type A and Accompanying Another Type of Liver Rupture—Type B
Rupture of the right hepatic lobe between its two sectors with a course parallel to the right triangular ligament-subtype 1	Type A subtype 1	Type B subtype 1
Longitudinal rupture of segment III or segment IV of the liver parallel to the falciform ligament-subtype 2	Type A subtype 2	Type B subtype 2
Rupture of the left hepatic lobe within segment II with a fissure parallel to the left coronary ligament of the liver-subtype 3	Type A subtype 3	Type B subtype 3

Table compiled on the basis of the original source [28].

**Table 2:** Annual distribution of identified cases by ICD-10 code and eligible liver injury cases by injury mechanism (2013–2025).

Year	Initial Cohort (N = 106)				Total Initial Cases	Final Study Cohort (N = 27)		Total Included Cases
	S36.1	S36.7	S36.8	S36.9		RTA	Falls	
2025	10	2	2	1	15	5	1	6
2024	5	4	1	0	10	0	0	0
2023	7	5 (1)	4 (1)	0	16 (2)	2	1	3
2022	6	0	4	0	10	3	0	3
2021	6	1	1	0	8	0	1	1
2020	4	2 (1)	0	0	6 (1)	2	1	3
2019	7	2	1	0	8	2	0	2
2018	3	0	0	0	3	1	0	1
2017	3	2 (1)	0	0	5 (1)	1	0	1
2016	5	2	1	0	8	3	0	3
2015	4	1	0	0	5	1	0	1
2014	6	1	1	0	8	2	1	3
2013	2	0	0	0	2	0	0	0
Total	68	22 (3)	15 (1)	1	106 (4)	22	5	27

S36.1—Injury of liver or gallbladder; S36.7—Injury of multiple intra-abdominal organs; S36.8—Injury of other intra-abdominal organs; S36.9—Injury of unspecified intra-abdominal organ; RTA—Road traffic accident.

**Table 3:** Demographic characteristics of the population and subpopulations included in the study.

Variable	Summary (*n* = 27)	Traffic Accidents (*n* = 22)	Falls from Height (*n* = 5)
Mean Age (SD)	35.9 (±12.4)	34.8 (±12.1)	40.2 (±11.8)
Median Age	35	34	40
Age Range (Min–Max)	19–59	19–59	20–49
Female, *n* (%)	10 (37.0%)	8 (36.4%)	1 (20%)
Male, *n* (%)	17 (63.0%)	14 (63.6%)	4 (80%)
Driver	-	7 (31.8%)	-
Passenger	-	6 (27.3%)	-
Pedestrian	-	6 (27.3%)	-
Motorcyclist	-	2 (9.1%)	-
N/A	-	1 (4.5%)	-

**Table 4:** Summary of physiological parameters and shock index in each group.

Group	Clinical Variable	Mean (SD)	Range	Median
Summary (*n* = 27)	GCS	9.7 (±5.4)	3–15	10
	SBP	106 (±38)	0–165	102
	HR	98 (±34)	0–140	100
	SI	0.89 (±0.58)	0.02–2.33	0.9
Traffic Accidents (*n* = 22)	GCS	8.5 (±5.5)	3–15	7.5
	SBP	98 (±34)	0–156	100
	HR	103 (±33)	0–140	100
	SI	1.13 (±0.44)	0.57–2.33	1.1
Falls from height (*n* = 5)	GCS	6.0 (±4.2)	3–14	4
	SBP	102 (±58)	0–165	124
	HR	75 (±43)	0–130	80
	SI	0.84 (±0.46)	0.48–1.63	0.63

GCS = Glasgow Coma Scale; SBP = Systolic blood pressure; HR = heart rate; SI = Shock Index (SI = HR/SBP).

**Table 5:** Classification of blunt liver injuries according to selected anatomical and clinical classifications.

No	Year	Age	Sex	Mech	Role	AAST OIS	WSES Grade	Arkuszewski Class	Ligament Site	Conte Class	Hardy Class	Compression	Comments	
					Or Height									
1	2025	35	M	RTA	Driver	III	II	2	Fal	-	-	-		
2	2025	29	M	RTA	Passenger	IV	IV	-	-	2	-	+		
3	2025	37	F	RTA	Pedestrian (Train)	IV	III	3	Lig	3	2	-	Book-like separation of the tissue	
4	2025	31	M	RTA	Motorcyclist	IV	III		-	2	-	+		
5	2025	22	M	RTA	Driver	IV	IV	1	Lig	2	-	-		
6	2023	42	F	RTA	Driver	IV	IV	-	-	-	-	-	Impossible to determine	
7	2023	37	F	RTA	Passenger	III	IV	-	-	3	-	-		
8	2022	36	F	RTA	Passenger	III	III	1	Lig	-	-	-		
9	2022	20	F	RTA	Passenger	III	IV	-	-	-	-	-	Impossible to determine	
					(Motorbike)									
10	2022	54	F	RTA	N/A	II	I	2	Fal, Lig	-	-	-		
11	2020	51	F	RTA	N/A	III	III	-	-	4	-	-		
12	2020	20	M	RTA	N/A	IV	IV	-	-	2	-	-		
13	2019	33	M	RTA	Pedestrian (Train)	V	IV	3	Lig	1; 3	2	+		
14	2019	47	F	RTA	Driver	V	IV	-	-	-	-	-		
15	2018	55	M	RTA	Pedestrian	III	IV	1	Lig	-	2	-		
16	2017	58	F	RTA	Passenger	V	III	-	-	-	-	-	Impossible to determine	
17	2016	27	M	RTA	Driver	II	IV	2	Fal	4	-	-	Bilateral rupture along falciform ligament	
18	2016	30	M	RTA	Driver	IV	IV	3	Lig	-	-	-		
19	2016	19	M	RTA	Pedestrian	IV	IV	-	-	-	2	-		
20	2015	21	F	RTA	Passenger	III	IV	-	-	4	-	-		
21	2014	59	M	RTA	Pedestrian	V	IV	3	Lig	-	-	-		
22	2014	21	M	RTA	N/A	III	II	3	Lig	-	-	-		
23	2025	42	M	Fall	10th floor	V	III	2; 3	Fal, Lig	3	2	+		
24	2023	40	F	Fall	4th floor	II	I	-	-	-	-	+	Impossible to determine; A chopped-up liver	
25	2021	49	M	Fall	7m	II	I	-	-	2	-	-		
26	2020	39	M	Fall	Scaffolding	III	IV	-	-	2	-	-	-	
27	2014	20	M	Fall	1st Floor	V	IV	1	Lig	-	-	-	-	

M—Male; F—Female; RTA—Road Traffic Accident; N/A—not applicable or not available; AAST OIS—American Association for the Surgery of Trauma Organ Injury Scale [21]; WSES Grade—World Society of Emergency Surgery Grade [16]; Arkuszewski Class—Classification recommended by P. Arkuszewski et al. (2021) [26]; Fal—Falciform; Lig—Ligamentous; Conte Class—Classification recommended by C. Conte (2012) [27]; Hardy Class—Classification recommended by K. J. Hardy (1972) [28].

**Table 6:** Clinical Characteristics of Trauma Patients Detailing Anatomical AIS Scores, Overall ISS, and Spectrum of Associated Injuries.

No	AIS1	AIS2	AIS3	AIS4	AIS5	AIS6	R/D	ISS	Associated Injuries
1	2	1	2	4	1	1	R	24	SI, mTBI
2	3	3	4	5	4	2	R	57	SI, Comm MFx, MRF, PC, PTX, Upper extremity Fx
3	3	1	3	4	1	1	R	34	Parietal bone Fx, SAH, MFx, MRF, PTX
4	3	0	2	5	0	1	R	38	Temporal bone Fx, Rib Fx, Clavicle Fx, PC
5	4	2	4	5	4	2	D	57	CFI, tPTX, SI, R. lower leg subamputation
6	3	0	4	4	5	1	D	57	Skull base Fx, MRF, Cardiac tamponade, bPTX, SI, multiple Pelvis & Extremity Fx
7	2	1	3	4	3	1	R	34	mTBI, PTX, SI, Complex Humerus Fx
8	1	1	4	4	4	1	R	48	Mandibular condyle Fx, MRF, FC, PC, PTX, SBMI, Pelvis Fx
9	0	0	2	4	0	1	R	21	Rib Fx, Scapula Fx, Small PTX
10	1	0	4	3	0	1	R	26	MRF, Sternal body Fx, Clavicle Fx, R. kidney pericapsular haematoma, Duodenal intramural haematoma, L1 Comp Fx
11	3	0	3	3	2	1	R	27	SAH, MRF, PC
12	3	0	4	4	4	2	D	48	TBI (pinpoint pupils), FC, PTX, PC, SI, RKI, GITP, Pelvis Fx, Humerus Fx, Knee instability
13	3	1	4	4	4	2	D	48	Occipital & Skull base Fx, FC, PTX, Clavicle Fx, SI, RKI, UP, Comm R. Femur Fx
14	5	1	3	5	2	1	D	59	CE, BSI, MRF, PTX, SI
15	3	2	3	3	3	1	R	27	Frontal bone Fx, PNC, MFx, MRF, PTX, Horseshoe kidney rupture, Extremity Fx
16	3	1	5	5	2	1	R	59	SAH, MRF, TAI, IVCI, SI
17	0	0	3	4	3	1	R	34	Rib Fx, PEff, PE, SI, SBMI, Pelvis injury
18	2	1	3	5	4	1	D	50	mTBI, Old brain haemorrhage foci, PTX, SI, Bilateral KI, UP, Extremity Fx
19	3	2	3	4	0	1	R	34	TBI, Mandible Fx, bPTX, PC, SI
20	0	0	3	5	3	1	R	43	Major SI, PC, LL, CSI
21	0	0	3	5	4	1	D	50	PC, R. PTX, Shattered R. kidney, Traumatic arm amputation
22	3	0	2	3	4	2	R	22	Pneumorrhachis, Rib Fx, SAH, UP, Acetabular Fx
23	4	0	4	5	5	1	D	66	Pelvis Fx, Femur Fx, Lower leg Fx, PTX
24	3	0	2	3	4	2	R	34	ICH, HTX, Pelvis Fx
25	0	0	2	3	3	1	R	22	PC, SI, CSI
26	4	1	5	4	4	1	R	57	EDH, Skull vault Fx, TAI, SI, Pelvis Fx, Hip dislocation
27	5	1	3	5	3	1	D	59	SCA, DI, SI, (6 for massive critical LI)

AIS1—Abbreviated Injury Scale Head and Neck injury; AIS2—Abbreviated Injury Scale Face injury; AIS3—Abbreviated Injury Scale Chest injury; AIS4—Abbreviated Injury Scale Abdomen injury; AIS5—Abbreviated Injury Scale extremity (including pelvis) injury; AIS6—Abbreviated Injury Scale External Injury; bPTX—Bilateral Pneumothorax; BSI—Brainstem Injury; CE—Cerebral Oedema; CFI—Craniofacial Injury; Comm—Comminuted; Comp—Compression; CSI—Cervical Spine Injury; DI—Diaphragmatic Injury; EDH—Epidural Haematoma; FC—Flail Chest; Fx—Fracture/Fractures; GITP—Gastrointestinal Tract Perforation; HTX—Hemothorax; ICH—Intracerebral Haemorrhage; ISS—Injury Severity Score; IVCI—Inferior Vena Cava Injury; KI—Kidney Injury; LI—Liver Injury; LL—Lung Laceration; MFx—Maxillofacial Fracture(s); MRF—Multiple Rib Fractures; mTBI—Mild Traumatic Brain Injury/Concussion; PC—Pulmonary Contusion; PE—Pulmonary Embolism; PEff—Pleural Effusion; PNC—Pneumocephalus; PTX—Pneumothorax; R.—Right; R/D—Recovery/Death; RKI—Right Kidney Injury; RTA—Road Traffic Accident; SAH—Subarachnoid Haemorrhage; SBMI—Small Bowel and Mesenteric Injury; SCA—Sudden Cardiac Arrest; SI—Splenic Injury; TAI—Thoracic Aortic Injury; TBI—Traumatic Brain Injury; tPTX—Tension Pneumothorax; UP—Unstable Pelvis.

**Table 7:** Prevalence and Characteristics of Associated Injuries, Categorised by Anatomical Region.

Anatomical Region	Specific Injuries
Abdominal	SI (*n* = 16), KI (*n* = 6), SBMI (*n* = 4), LI (*n* = 1), IVCI (*n* = 1)
Chest	PTX (*n* = 15), MRF (*n* = 13), PC (*n* = 9), Shoulder/Sternal Fx (*n* = 4), FC (*n* = 3), TAI (*n* = 2), Other (*n* = 6)
Head & Brain	Skull Fx (*n* = 6), mTBI/TBI (*n* = 5), SAH (*n* = 4), ICH/EDH (*n* = 2), CE/BSI (*n* = 2), PNC (*n* = 1)
Maxillofacial	MFx, Comm MFx, CFI, Mandibular Fx (Total *n* = 6)
Pelvis & Extremities	Pelvic Fx/UP (*n* = 10), Lower Limb (*n* = 5), Upper Limb (*n* = 4), Extremity Fx (*n* = 3), Amputation (n = 2)
Spine	CSI (*n* = 2), L1 Comp Fx (*n* = 1), Pneumorrhachis (*n* = 1)

BSI—Brainstem Injury; CE—Cerebral Oedema; CFI—Craniofacial Injury; Comm—Comminuted; Comp—Compression; CSI—Cervical Spine Injury; DI—Diaphragmatic Injury; EDH—Epidural Haematoma; FC—Flail Chest; Fx—Fracture/Fractures; GITP—Gastrointestinal Tract Perforation; HTX—Hemothorax; ICH—Intracerebral Haemorrhage; IVCI—Inferior Vena Cava Injury; KI—Kidney Injury; LI—Liver Injury; LL—Lung Laceration; MFx—Maxillofacial Fracture(s); MRF—Multiple Rib Fractures; mTBI—Mild Traumatic Brain Injury; PC—Pulmonary Contusion; PE—Pulmonary Embolism; PEff—Pleural Effusion; PNC—Pneumocephalus; PTX—Pneumothorax; RKI—Right Kidney Injury; SAH—Subarachnoid Haemorrhage; SBMI—Small Bowel and Mesenteric Injury; SCA—Sudden Cardiac Arrest; SI—Splenic Injury; TAI—Thoracic Aortic Injury; TBI—Traumatic Brain Injury; bPTX—Bilateral Pneumothorax; tPTX—Tension Pneumothorax; UP—Unstable Pelvis.

**Table 8:** The comparison of preoperative computed tomography scan results with intraoperative diagnosis in patients with traumatic liver injuries.

No	CT Pre	CT Result Original	Intraoperative Diagnosis	Haematoma	Rupture	Contusion	Tearing/Splitting	No Changes	Suspected Rupture	Suspected Haematoma	Post-Traumatic Disorders	CT After	CT not Performed
1	+	Liver parenchyma contusion	Rupture of liver parenchyma	−	−	+	−	−	−	−	−	−	−
2	+	Rupture of capsule and parenchyma	Rupture of the capsule and parenchyma	−	+	−	−	−	−	−	−	−	−
3	+	Post-traumatic changes?	Liver parenchyma rupture	−	−	−	−	−	−	−	+	−	−
4	−	Parenchyma contusion, status post-surgery	Tearing of the liver capsule and parenchyma	−	−	+	−	−	−	−	−	−	−
5	−	Status post liver rupture	Liver parenchyma rupture	−	+	−	−	−	−	−	−	−	−
6	+	Nothing	Liver parenchyma rupture	−	−	−	−	+	−	−	−	−	−
7	+	Liver haematoma	Liver parenchyma rupture	+	−	−	−	−	−	−	−	−	−
8	+	Suspected liver rupture, haematoma	Liver parenchyma rupture	+	−	−	−	−	+	−	−	−	−
9	+	Suspected liver rupture and contusion	Liver parenchyma rupture	−	−	+	−	−	+	−	−	−	−
10	?	NO DATA	rupture of the liver capsule	−	−	−	−	−	−	−	−	−	+
11	+	Suspected liver haematoma	Liver parenchyma rupture	−	−	−	−	−	−	+	−	−	−
12	−	Liver contusion and rupture	Liver contusion and rupture	−	+	+	−	−	−	−	−	−	−
13	−	Status post packing	liver dissection	−	−	−	−	−	−	−	−	−	−
14	−	Status post packing	Liver parenchyma rupture	−	−	−	−	−	−	−	−	−	−
15	+	Liver contusion and rupture	NO DATA ON THE OPERATION	−	+	+	−	−	−	−	−	−	−
16	+	Liver contusion and rupture	Liver parenchyma rupture	−	−	+	−	−	−	−	−	−	−
17	+	NO DATA	Liver parenchyma rupture	−	−	−	−	−	−	−	−	−	+
18	−	Liver contusion and rupture	Liver parenchyma rupture	−	+	+	−	−	−	−	−	−	−
19	−	Tear of liver capsule and parenchyma	liver dissection	−	+	−	+	−	−	−	−	−	−
20	+	Liver parenchyma rupture	Liver parenchyma rupture	−	+	−	−	−	−	−	−	−	−
21	+	Nothing	Liver parenchyma rupture	−	−	−	−	+	−	−	−	−	−
22	+	Liver parenchyma contusion	Liver parenchyma rupture	−	−	+	−	−	−	−	−	−	−
23	−	−	deep laceration of the liver	−	−	−	−	+	−	−	−	−	−
24	+	Parenchyma contusion	rupture of the liver capsule	−	−	+	−	−	−	−	−	−	−
25	+	Suspected parenchyma contusion	Liver parenchymal contusion	−	−	+	−	−	−	−	+	+	−
26	+	Suspected parenchyma contusion	Liver parenchyma rupture	−	−	+	−	−	−	−	+	+	−
27	−	−	liver dissection	−	−	−	+	+	−	−	−	−	−

F—Female; M—Male; CT—Computed Tomography; CT pre—Computed Tomography pre-surgery; CT ps—Computed Tomography post-surgery; +—Yes; −—No; ?—Unknown.

**Table 9:** Evaluation of the diagnostic compliance and sensitivity of preoperative computed tomography with intraoperative verification in traumatic liver injuries.

	CT Result/Facts	For the Entire Group N = 27 (%)	With Regard to the Subgroup *n*/N (%)	Interpretation and Clinical Significance
CT scan (N = 27)	A CT scan was performed preoperatively.	17 (63.0%)		Main group for further radiological assessment.
	No CT scan (emergency surgery)	4 (14.8%)		Patients too unstable for diagnosis.
	CT after surgery/no data available	5 (18.5%)		Diagnostics performed in the post-operative mode.
	Status unknown	1 (3.7%)		Incomplete documentation.
Compliance (N = 14)	Total compliance	2/27 (7.4%)	2/14 (14.3%)	The CT scan accurately described the fracture confirmed during surgery.
	Partial compliance	3/27 (11.1%)	3/14 (21.4%)	The CT scan indicated a suspicion without a definite diagnosis.
	Underestimation	9/27 (33.3%)	9/14 (64.3%)	The CT scan drastically underestimated the actual extent of the injury.
Type of discrepancy (N = 9)	The contusion turned out to be a rupture	5/27 (18.5%)	5/9 (55.6%)	The most common mistake: milder condition on CT scan, severe during surgery.
	The haematoma turned out to be a rupture	2/27 (7.4%)	2/9 (22.2%)	Post-traumatic changes were described instead of active damage.
	No changes turned out to be a rupture	2 (7.4%)	2/9 (22.2%)	

**Table 10:** Spearman’s rank-correlation results (Risk factor for death).

Comparison Variables		ρ	*p*-Value	Notes/Interpretations
Death	AAST OIS	0.70	<0.001	The strongest predictor in the study group. A higher degree of liver damage is associated with a higher risk of death.
Death	GCS	−0.65	<0.001	The greater the depth of the coma, the greater the risk of death.
Death	SI	0.50	<0.001	Greater instability increases the risk of death (although less than AAST OIS)
Death	LOS	−0.74	<0.001	Deaths occur earlier (short LOS). Survival is associated with prolonged hospitalisation
AAST	GCS	−0.74	<0.001	Severe liver damage is accompanied by severe disturbances of consciousness (multiple organ injury or shock)

ρ—Spearman’s correlation coefficient; GCS—Glasgow Coma Scale; SI—Shock Index (SI = HR/SBP); LOS—Length of Stay; AAST OIS—American Association for the Surgery of Trauma Organ Injury Scale.

## Data Availability

The primary data presented in this study are available within the article. Additional raw data supporting the findings of this research are available from the corresponding author upon reasonable request.

## Abbreviations

The following abbreviations are used in this manuscript:

+	Yes
−	No
AAST OIS	American Association for the Surgery of Trauma Organ Injury Scale [21]
AIS1	Abbreviated Injury Scale Head and Neck injury
AIS2	Abbreviated Injury Scale Face injury
AIS3	Abbreviated Injury Scale Chest injury
AIS4	Abbreviated Injury Scale Abdomen injury
AIS5	Abbreviated Injury Scale extremity (including pelvis) injury
AIS6	Abbreviated Injury Scale External Injury
Arkuszewski Class	Classification recommended by P. Arkuszewski et al. (2021) [26]
AT	Abdominal Trauma
BLI	Blunt Liver Injury
BLT	Blunt Abdominal Trauma
Bpm	Beats per minute
bPTX	Bilateral Pneumothorax
BSI	Brainstem Injury
CE	Cerebral Oedema
CFI	Craniofacial Injury
Comm	Comminuted
Comp	Compression
Conte Class	Classification recommended by C. Conte (2012) [27]
CSI	Cervical Spine Injury
CT	Computed Tomography
CT pre	Computed Tomography pre-surgery
CT ps	Computed Tomography post-surgery
DCS	Damage Control Surgery
DI	Diaphragmatic Injury
DBP	Diastolic Blood Pressure
ED	Emergency Department
EDH	Epidural Haematoma
eFAST	Extended Focused Assessment with Sonography for Trauma
F	Female
Fal	Falciform
FAST	Focused Assessment with Sonography for Trauma
FC	Flail Chest
Fx	Fracture/Fractures
GCS	Glasgow Coma Scale
GITP	Gastrointestinal Tract Perforation
Hardy Class	Classification recommended by K. J. Hardy (1972) [28]
HR	heart rate
HTX	Hemothorax
ICH	Intracerebral Haemorrhage
ISS	Injury Severity Score
IVCI	Inferior Vena Cava Injury
KI	Kidney Injury
LI	Liver Injury
Lig	Ligamentous
LL	Lung Laceration
LOS	Length of Stay
M	Male
MFx	Maxillofacial Fracture(s)
MRF	Multiple Rib Fractures
mTBI	Mild Traumatic Brain Injury/Concussion
N/A	Not applicable or not available
NOM	Non-operative management
OIS	Organ Injury Scale
PC	Pulmonary Contusion
PE	Pulmonary Embolism
PEff	Pleural Effusion
PMCOT	Provincial Multispecialist Centre for Oncology and Traumatology
PNC	Pneumocephalus
PTX	Pneumothorax
R.	Right
R/D	Recovery/Death
RKI	Right Kidney Injury
RTA	Road traffic accident
S36.1	Injury of liver or gallbladder
S36.7	Injury of multiple intra-abdominal organs
S36.8	Injury of other intra-abdominal organs
S36.9	Injury of unspecified intra-abdominal organ
SAH	Subarachnoid Haemorrhage
SBMI	Small Bowel and Mesenteric Injury
SBP	Systolic blood pressure
SCA	Sudden Cardiac Arrest
SI	Shock Index, Allgower’s Index (SI = HR/SBP)
TAI	Thoracic Aortic Injury
TBI	Traumatic Brain Injury
tPTX	Tension Pneumothorax
UP	Unstable Pelvis
WSES	World Society of Emergency Surgery [16]
ρ	Spearman’s correlation coefficient
